# SIBLING PAIR ANALYSIS IN THE IDENTIFICATION PROCESS OF THE MADURESE POPULATION USING STR CODIS LOCI

**DOI:** 10.21010/Ajid.v16i2.5

**Published:** 2022-05-06

**Authors:** Ahmad Yudianto, Arofi Kurniawan, Fery Setiawan, Egita Windrianatama Puspa, Racy Youngest

**Affiliations:** 1 Department of Forensic Medicine and Medicolegal, Faculty of Medicine, Universitas Airlangga, Indonesia; 2 Department of Forensic Odontology, Faculty of Dental Medicine, Universitas Airlangga, Indonesia; 3 Forensics Study Program, Magister Program of Postgraduate School, Universitas Airlangga, Indonesia; 4 Human Genetics Study Group, Institute of Tropical Diseases, Universitas Airlangga, Indonesia; 5 Doctoral Program of Medical Science, Faculty of Medicine, Universitas Airlangga, Indonesia

**Keywords:** Identification, STR CODIS, Siblings, Madurese Population, Human &, Mortality

## Abstract

**Background:**

The most common problem in forensic personal identification, particularly in paternity tests, is the availability of information originating from a mother or a father that can be used as a comparison in the forensic DNA examination process. The use of sibling analysis in paternity tests is still not widely known.

**Materials and methods:**

The respondents of this study were 25 families of the Madurese population with 6, 9, and 10 sibling pairs categorized as female-female, male-male, and male-female siblings. The kinship relationship was analyzed by using 13 STR CODIS loci technique (TPOX, D3S1358, FGA, D5S818, CSFIPO, D7S820, D8S1179, THOI, vWA, D13S317, D16S539, D18S51, D21S11), and Amelogenin (Amel) x: 106bp,y: 112bp).

**Results:**

The results of DNA contents and purity examination of the extracted DNA sample showed that the average value of DNA contents was 675±5.35 ng/μL with a purity range of 1.05-1.86. The findings proved that the male-male siblings of the Madurese population had the highest allele sharing percentage at the loci of D13S317, D16S539, and D21S11. The highest allele sharing percentage for female-female siblings was at the loci of TPOX and D21S11. Meanwhile, the male-female siblings had the highest allele sharing percentage at the loci of TPOX, D5S818, vWA, D7S820, THO1, vWA, and D13S317.

**Conclusion:**

The main STR loci recommended in the male-female siblings of Madurese population identification are TPOX, D13S317, and D21S11.

## Introduction

A paternity test is a means to determine whether a man is a biological father. It is a valid legal procedure for determining paternity. Paternity determination is very complicated because many aspects need to be examined. Until now, the solution for paternity problems is assessed by examining the similar or dissimilar features of a child and the alleged father. The similar features here refer to characteristics of irises’ color of the eyes, hair, unique manners or way of speaking, and stature (Yudianto, 2015: Yudianto et al., 2021).

The most common challenge faced during the paternity test process is the unavailability of genetic information from the parents. The unavailability of genetic information from both parents as a comparison in an identification process is one of the problems in forensic DNA analysis, particularly in paternity tests (Sykuriani, 2012: Yudianto, 2013). This condition requires a comparison that has a close family line as a solution in the process of forensic DNA analysis such as biological siblings (Gaytmenn et al., 2002).

The forensic identification process of siblings is also conducted through the examination of alleles at the STR loci of nuclear DNA. Based on this reason, the use of siblings as the comparison through STR remains unknown in Indonesia as well as other countries regardless of the demand for ethnic and population diversity, natural disasters, and other events that require forensic readiness. The application usually targets the use of human profiling and typing, forensic application, sibship relationship, craniology tracing or individual identification, and incestuous identification (Consetino et al., 2015: Karbeyas K et al., 2016; Yudianto et al., 2021).

The Madurese are one of the ethnic groups with the highest population in Indonesia. Based on National Census 2010, the Madurese population was 7,179,356. They are originating from the island of Madura and its nearby islets (Puteran, Gili Iyang, Sapudi, Gili Raja, Gili Genting, Raas, etc). Additionaly, they are a wandering tribe that can be found in many places across Indonesia. The population can also be found in neighboring countries, such as Malaysia and Singapore.

In general, the Madurese population was chosen in this study because of their unique characteristics in terms of their temperament and unique accents. Most of the Madurese population have strong work ethics and are adventurous. These characteristics make Madurese people tend to migrate from their homeland (Prastowo W et al, 2018; Sosiawan A et al., 2019).

## Materials and methods

### Population and Research Sample

The population of this research was all subjects undergoing paternity examination at Human Genetic Study Group, The Institute of Tropical Disease, Universitas Airlangga. The research sample was originating from the peripheral blood of paternity examination respondents, consisting of a father, a mother, and two children of the Madurese population. The father and the mother serve as the control/reference for allele sharing between the siblings. The inclusion criterion for this research was a family consisting of at least three generations of pure Madurese population with two biological children. This research has obtained ethical clearance from the Faculty of Dentistry Universitas Airlangga Number 275/HRECC.FODM/VI/2020. The number of respondents in this study was 25 with 100 samples. This research was conducted at the Human Genetic Study Group, the Institute of Tropical Disease Universitas Airlangga.

### Sample Preparation

One hundred peripheral blood samples of the respondents were stored inside tubes and labeled as A [father], B [mother], and C [children]. The labels were used to indicate that the samples were collected from fathers, mothers, and biological children.

### DNA Extraction

The DNA extraction process of 100 peripheral blood samples was carried out using the DNAzol method (McClintock 2014). 50 μL of distilled water was added to the isolated DNA pellet.

### DNA Amplification

The DNA amplification process was conducted through Polymerase Chain Reaction (PCR) process (PowerPlex® 21Systems, Promega, USA) targeting specific DNA sequences to replicate the isolated DNA. The amplification of 80 samples was conducted using 13 Short Tandem Repeats [STR]-Combined DNA Index System [CODIS] (TPOX, D3S1358, FGA, D5S818, CSFIPO, D7S820, D8S1179, THOI, vWA, D13S317, D16S539, D18S51, D21S11) and Amelogenin (Amel) x:106bp,y:112bp. The amplification adjustment for D3S1358, FGA, D8S1179, D18S51, and D21S11 was: 96 °C- 2 minutes, followed by [ 94 °C-1 minute, 60 °C-1 minute, 70 °C-1.5 minutes for 10 cycles], [90 °C-1 minute, 64 °C- 1 minute, 70 °C-1.5 minutes for 25 cycles]. The adjustment for CSF1PO was 96 °C-2 minutes, followed by [94 °C-1 minute, 64 °C-1 minute, 70 °C-1.5 minutes for 10 cycles], [90 °C-1 minute, 64 °C- 1 minute, 70 °C-1.5 minutes for 30 cycles]. The amplification adjustment for D5S818, D7S820, and D13S317 was 96 °C- 1 minute, followed by [94 °C-30 seconds, 60 °C-30 seconds, 70 °C-45 seconds for 10 cycles], [90 °C-30 seconds, 64 °C- 30 seconds, 70 °C-45 seconds for 30 cycles]. The amplification adjustment for D16S539 was 96 °C-1 minute, followed by [94 °C-1 minute, 59 °C-1 minutes, 72 °C-1.5 minutes for 25 cycles], and 72 °C-1 minutes. All DNA templates were stored at 4 °C temperature (Promega corp, 2001).

### Electrophorese Gel

**Figure 1:**
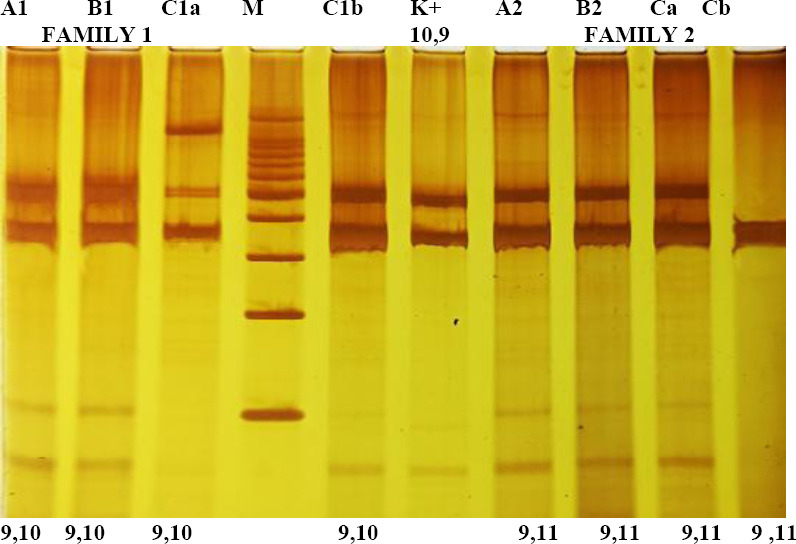
PCR visualization of CSF1PO locus [321bp-357bp]. M (Marker), A (Father), B (Mother), Ca (Child 1), Cb (Child 2)

The visualization of PCR results was conducted through vertical electrophoresis using 6% polyacrylamide agarose gel [PAGE] [Bio-Rad Mini-PROTEAN®] with silver nitrate staining (Figure 1).

### Sample Alleles

**Figure 2:**
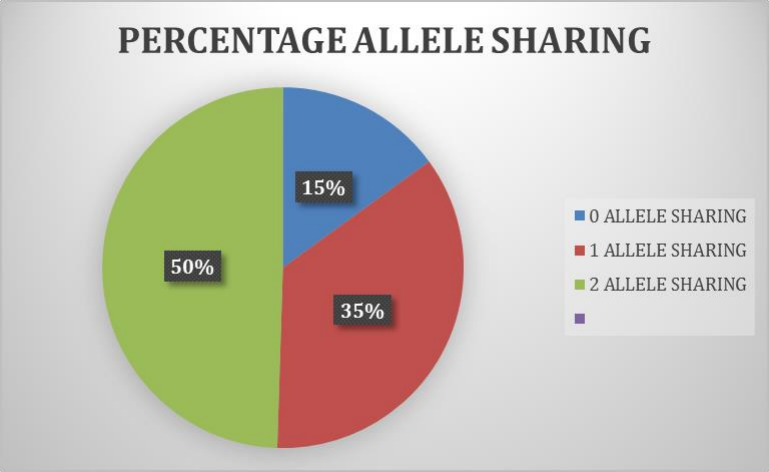
Allele Sharing Percentage

**Figure 3:**
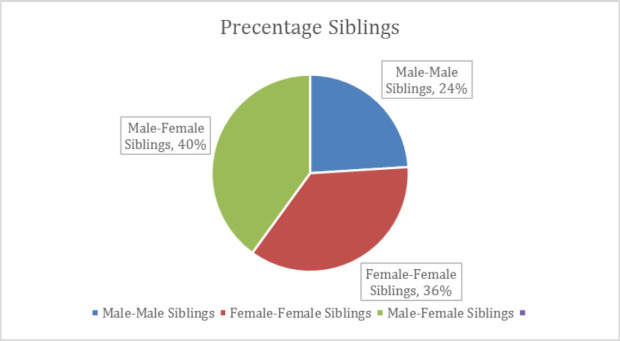
Sibling Percentage Based on Sex

**Figure 4:**
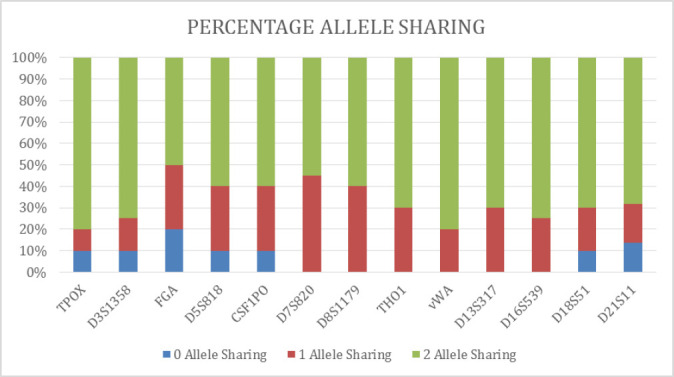
Percentage of Allele Sharing on Siblings

**Figure 5:**
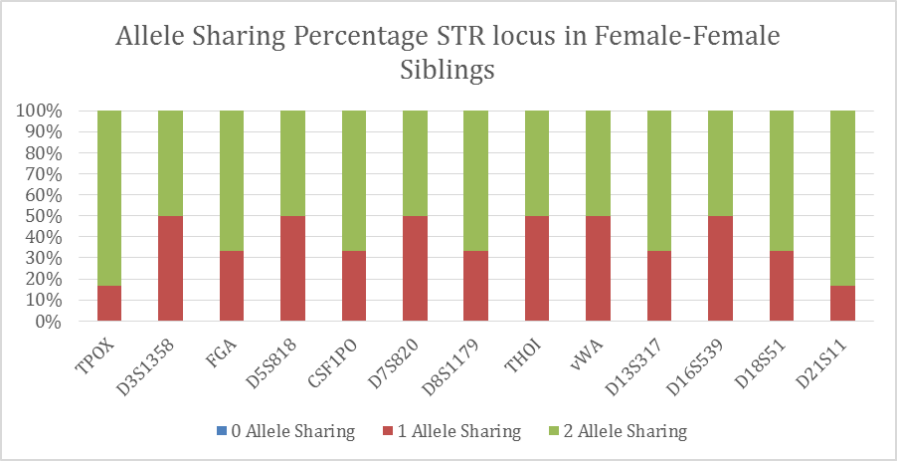
Percentage of STR loci allele sharing on female-female siblings

The reading of PCR visualization on the electrophoresis gel resulted in alleles at each locus with K562 as the control (Table I). The alleles were matched with family members (the father, the mother, and the children) and the value of allele frequency (Table II). The analysis was based on the frequency of allele sharing of kinship analysis between biological siblings at every STR CODIS locus by examining the allele sharing (Figure 2, 3, 4, 5).

**Table 1:** STR Allele Profile of the 25 Families

		STR CODIS													

Family	Code	TPOX	D3 S1358	FGA	D5 S818	CSF1P0	D7 S820	D8 S1179	THO1	vWA	D13 S317	D16 S539	D18 S51	D21S11	Amel
1	A1	8,9	16,17	21,24	11,12	9,10	9,11	12,13	8,9	15,18	8,8	11,11	15,16	29,31	106,112
	B1	8,10	16,16	21,24	11,12	8,10	9,11	12,13	9,10	16,17	8,8	11,11	15,16	30,31	106
	C1a	8,8	16,16	21,21	12,12	8,10	9,11	12,12	9,10	15,17	8,8	11,11	15,16	30,31	106,112
	C1b	8,9	16,16	21,24	11,12	9,10	9,11	12,13	9,10	15,17	8,8	11,11	15,15	31.31	106,112
2	A2	9,10	15,17	21,22	10,12	9,10	8,8	11,12	11,13	19,20	8,9	10,11	14,15	29,31	106,112
	B2	8,9	16,17	21,24	9,10	8,9	9,10	10,11	9,10	18,19	8,10	11,13	15,15	30,31	106
	C2a	8,9	17,17	21,21	10,10	9,9	8,9	11,11	9,11	19,19	8,8	11,11	15,15	31,31	106
	C2b	8,9	15,17	21,22	10,12	8,9	8,9	11,11	9,11	19,19	8,9	11,11	15,15	31,31	106
3	A3	8,9	15,17	20,24	9,11	8,8	8,9	9,10	8,9	15,17	8,11	9,11	14,16	29,32	106,112
	B3	9,10	16,17	22,24	11,11	9,10	9,10	11,12	8,10	18,19	9,10	9,10	16,16	30,32	106
	C3a	9,10	16,17	24,24	11,11	8,9	9,9	10,11	8,8	17,18	9,11	9,9	16,16	32.32	106
	C3b	9,10	16,17	24,24	11,11	8,9	8,9	10,12	8,9	17,18	8,9	9,9	16,16	29,30	106
4	A4	9,9	16,18	22,24	8,9	9,10	8,11	9,12	11,13	20,21	9,11	10,11	15,16	31,32	106,112
	B4	9,10	17,18	21,22	10,12	9,11	9,10	12,13	11,12	16,18	9,11	10,10	16,17	29,31	106
	C4a	9,9	18,18	22,22	9,10	9,9	9,11	12,12	11,11	16,20	9,9	10,10	16,16	31,31	106,112
	C4b	9,9	18,18	21,24	9,10	9,11	8,9	12,12	11,11	16,20	9,9	10,10	16,16	31,31	106
5	A5	8,10	16,17	20,21	11,13	8,12	9,11	10,12	8,12	16,17	9,11	9,11	14,17	29,31	106,112
	B5	8,9	17,17	22,24	9,11	9,10	9,11	9,12	9,11	18,20	11,11	8,10	16,17	30,31	106
	C5a	8,8	17,17	21,22	11,11	9,12	9,9	12,12	8,9	16,18	11,11	8,9	14,16	31,31	106
	C5b	8,8	17,17	21,22	11,11	9,12	9,9	12,12	8,9	16,18	11,11	8,9	17,17	31,31	106
6	A6	7,10	15,18	21,21	9,11	8,9	8,10	11,12	8,9	19,20	8,9	11,13	14,15	29,31	106,112
	B6	10,11	16,17	22,23	11,11	10,11	9,11	12,13	10,13	18,19	10,12	10,12	15,15	30,31	106
	C6a	7,11	16,18	21,22	11,11	8,11	8,9	12,12	8,10	19,19	9,10	11,12	15,15	30,31	106,112
	C6b	10,11	16,18	21,23	11,11	8,11	9,10	12,12	8,10	19,19	9,10	11,12	15,15	31.31	106,112
7	A7	9,10	16,17	20,21	10,12	10,11	8,11	11,13	11,12	17,19	8,8	11,11	14,16	29,31	106,112
	B7	8,9	15,17	22,24	11,13	9,10	9,10	10,12	9,10	17,20	8,9	10,10	16,16	30,31	106
	C7a	8,9	17,17	21,22	11,12	10,10	9,11	11,12	9,11	17,17	8,8	10,11	16,16	31,31	106
	C7b	8,9	15,17	21,22	11,12	9,11	8,9	11,12	9,11	17,19	8,8	10,11	16,16	31,31	106,112
8	A8	9,10	17,19	22,24	10,12	9,11	9,11	12,13	11,12	15,17	8,9	9,11	14,17	31,32	106,112
	B8	8,9	16,18	21,23	9,11	10,11	9,11	12,13	10,11	20,21	8,10	8,10	16,17	29,31	106
	C8a	8,9	17,18	21,24	9,10	9,11	9,9	12,12	11,11	15,21	8,8	8,9	14,16	31,31	106,112
	C8b	8,9	17,18	21,24	9,10	9,11	9,9	12,13	11,11	15,21	8,9	8,9	17,17	31,31	106,112
9	A9	11,13	16,18	22,23	11,12	8,10	9,11	11,12	8,13	16,17	9,11	11,13	14,15	29,31	106,112
	B9	11,12	17,17	21,24	10,11	9,10	9,11	12,13	9,10	18,20	9,11	10,12	15,15	30,31	106
	C9a	11,11	16,17	21,24	11,11	8,9	9,9	12,12	8,9	16,18	9,9	11,12	15,15	31,31	106
	C9b	11,11	16,17	21,24	11,11	8,9	9,9	12,12	8,9	16,18	9,9	11,12	15,15	31,31	106,112
10	A10	10,11	15,17	20,24	9,11	8,12	9,11	11,13	8,11	15,18	9,11	10,11	15,16	31,32	106,112
	B10	9,10	16,18	19,22	11,13	8,10	11,11	10,12	8,9	16,17	11,11	10,10	16,17	29,31	106
	C10a	10,10	15,18	20,22	11,11	8,8	11,11	11,12	8,8	15,17	11,11	10,10	16,16	31,31	106,112
	C10b	9,11	16,17	20,22	9,13	8,8	11,11	11,12	8,8	15,17	11,11	10,10	16,16	31,31	106,112
11	A11	11,13	15,17	19,21	10,12	9,10	8,8	12,13	8,9	15,17	9,11	9,11	14,17	29,31	106,112
	B11	11,12	16,18	21,23	9,11	9,11	8,8	12,13	10,13	18,19	11,11	8,10	16,17	30,31	106
	C11a	11,11	15,18	21,21	9,10	9,9	8,8	12,12	8,10	17,18	11,11	8,9	14,16	31,31	106,112
	C11b	11,11	16,17	21,21	9,10	9,11	8,8	12,13	8,10	17,18	11,11	8,9	17,17	31,31	106
12	A12	11,13	16,17	20,24	10,12	9,11	8,9	12,13	11,12	20,21	8,8	11,13	14,15	29,31	106,112
	B12	11,12	16,16	19,22	9,11	10,11	8,10	12,13	9,10	16,18	8,8	10,12	15,15	30,31	106
	C12a	11,11	16,16	20,22	9,10	9,11	8,8	12,12	9,11	16,20	8,8	11,12	15,15	30,31	106
	C12b	11,11	16,16	20,22	9,10	9,11	8,9	12,13	9,11	16,20	8,8	11,12	15,15	31.31	106
13	A13	9,10	16,17	22,24	10,12	8,10	8,11	11,12	8,13	15,18	8,9	10,11	15,16	29,31	106,112
	B13	8,9	17,17	21,23	9,10	9,10	9,10	12,13	9,10	16,17	8,10	10,10	16,17	30,31	106
	C13a	8,9	17,17	21,24	10,10	8,9	9,11	12,12	8,9	15,17	8,8	10,10	16,16	31,31	106,112
	C13b	8,9	17,17	21,24	10,12	8,9	8,9	12,12	8,9	15,17	8,9	10,10	16,16	31,31	106,112
14	A14	8,9	15,18	22,23	9,11	8,12	9,11	11,13	8,11	16,17	8,8	9,11	14,17	29,31	106,112
	B14	9,10	16,17	21,24	11,11	8,10	9,11	10,12	8,9	18,20	8,8	8,10	16,17	30,31	106
	C14a	9,10	16,18	21,24	11,11	8,8	9,9	11,12	8,8	16,18	8,8	8,9	14,16	30,31	106,112
	C14b	9,10	16,18	21,24	11,11	8,8	9,9	11,12	8,8	16,18	8,8	8,9	17,17	31.31	106
15	A15	9,9	17,19	21,24	8,9	9,10	9,11	12,13	11,13	15,18	9,11	9,11	14,17	28,30	106,112
	B15	9,10	16,18	21,24	10,12	9,11	11,11	12,13	11,12	16,17	9,11	8,10	16,17	30,31	106
	C15a	9,9	17,18	21,21	9,10	9,9	11,11	12,12	11,11	15,17	9,9	8,9	14,16	30,30	106,112
	C15b	9,9	17,18	21,24	9,10	9,11	11,11	12,13	11,11	15,17	9,9	8,9	17,17	30,31	106
16	A16	9,10	16,18	21,22	8,9	8,12	8,9	11,12	8,12	19,20	9,11	10,11	15,16	31,32	106,112
	B16	8,9	17,17	21,24	10,12	8,10	8,10	10,11	9,11	18,19	11,11	10,10	16,17	29,31	106
	C16a	8,9	16,17	21,21	9,10	8,8	8,8	11,11	8,9	19,19	11,11	10,10	16,16	31,31	106,112
	C16b	8,9	16,17	21,22	9,10	8,8	8,9	11,11	8,9	19,19	11,11	10,10	16,16	31,31	106,112
17	A17	11,13	15,17	20,24	10,12	9,10	9,11	9,10	11,13	17,19	8,8	9,11	14,17	29,31	106,112
	B17	11,12	16,18	19,22	9,10	9,11	9,11	11,12	9,10	17,20	8,8	8,10	16,17	30,31	106,
	C17a	11,11	15,18	20,22	10,10	9,9	9,9	10,11	9,11	17,17	8,8	8,9	14,16	31,31	106
	C17b	11,11	16,17	20,22	10,12	9,11	9,9	10,12	9,11	17,19	8,8	8,9	17,17	31,31	106,112
18	A18	10,11	16,17	22,24	8,9	8,12	9,11	11,13	8,9	16,17	8,9	11,13	14,15	29,31	106,112
	B18	9,10	16,16	21,23	10,12	9,10	11,11	10,12	9,10	18,20	8,10	10,12	15,15	30,31	106
	C18a	10,10	16,16	21,24	9,10	9,12	11,11	11,12	9,10	16,18	8,8	11,12	15,15	30,31	106,112
	C18b	9,11	16,16	21,24	9,10	9,12	11,11	11,12	9,10	16,18	8,9	11,12	15,15	31.31	106,112
19	A19	11,13	15,18	21,24	9,11	8,12	9,11	12,13	11,13	19,20	8,11	9,11	14,17	31,32	106,112
	B19	11,12	16,17	21,24	11,13	8,10	11,11	12,13	9,10	18,19	9,10	8,10	16,17	29,31	106
	C19a	11,11	16,18	21,21	11,11	8,8	11,11	12,12	9,11	19,19	9,11	8,9	14,16	31,31	106
	C19b	11,11	16,18	21,24	9,13	8,8	11,11	12,13	9,11	19,19	8,9	8,9	17,17	31,31	106,112
20	A20	9,9	17,19	21,22	10,12	9,10	8,8	11,12	8,13	17,19	8,9	11,13	14,15	29,31	106,112
	B20	9,10	16,18	21,24	9,11	9,11	8,8	10,11	9,10	17,20	8,10	10,12	15,15	30,31	106
	C20a	9,9	17,18	21,21	9,10	9,9	8,8	11,11	8,9	17,17	8,8	11,12	15,15	31,31	106,112
	C20b	9,9	17,18	21,22	9,10	9,11	8,8	11,11	8,9	17,19	8,8	11,12	15,15	31,31	106,112
21	A20	8,9	17,18	21,22	10,11	9,10	8,8	11,11	8,11	17,19	8,9	11,11	14,15	29,30	106,112
	B20	9,10	16,18	21,24	9,11	9,11	7,8	10,11	9,10	17,20	8,10	10,12	15,15	30,31	106
	C20a	9,9	17,18	21,21	9,10	9,9	8,8	11,11	8,11	17,17	8,8	11,12	15,15	30,31	106
	C20b	9,9	17,18	21,22	9,11	9,11	7,8	11,11	8,8	17,19	8,10	11,12	15,15	31,31	106
22	A20	11,13	17,17	21,24	10,12	9,10	7,8	11,12	8,13	17,18	8,9	11,13	14,15	29,31	106,112
	B20	11,12	16,18	21,24	9,11	9,11	8,8	10,11	9,11	17,19	8,10	10,12	15,15	30,31	106
	C20a	11,11	17,18	21,21	9,10	9,9	7,8	11,11	8,9	17,17	8,9	11,12	15,15	30,31	106,112
	C20b	11,11	17,18	21,24	9,10	9,11	8,8	11,11	8,9	17,19	8,8	11,12	15,14	31,31	106
23	A20	10,11	17,19	20,22	10,12	9,10	8,8	11,12	8,9	17,20	8,11	11,12	14,15	29,31	106,112
	B20	9,10	16,18	20,23	9,11	9,11	8,8	10,11	9,10	17,20	8,10	10,12	15,15	30,31	106
	C20a	10,10	17,18	20,20	9,10	9,9	8,8	11,11	8,9	17,17	8,8	11,12	15,15	31,31	106,112
	C20b	9,11	17,18	20,22	9,10	9,11	8,8	11,11	8,9	17,20	8,11	11,12	15,15	31,31	106,112
24	A20	9,11	16,19	21,23	10,12	10,10	8,8	11,11	8,13	17,19	8,9	11,12	14,15	29,30	106,112
	B20	9,10	16,18	21,24	9,11	9,11	8,8	10,10	9,10	18,20	8,10	10,12	15,16	30,31	106
	C20a	9,9	16,18	21,23	9,10	9,10	8,8	11,10	8,9	17,18	8,8	11,12	15,16	30,31	106
	C20b	9,9	16,18	21,21	9,10	9,10	8,8	11,10	8,9	17,18	8,8	11,12	14,15	30,30	106,112
25	A20	9,10	18,19	21,22	10,11	9,10	8,8	11,12	8,11	17,19	8,11	11,13	15,15	29,30	106,112
	B20	9,10	18,18	21,23	9,10	9,11	8,8	10,12	9,10	17,20	8,10	10,11	15,15	30,31	106
	C20a	9,10	18,18	21,21	9,10	9,9	8,8	11,12	8,8	17,17	8,8	11,11	15,15	29,30	106
	C20b	10,10	18,18	21,22	9,10	9,10	8,8	12,12	8,10	17,19	8,11	11,11	15,15	30,31	106

**Table 2:** Frequencies of STR Alleles of the Sample (N=200)

Allele	Frequencies	Allele	Frequencies
**TPOX**		**THOI**	
7	0,01250	8	0,25250
8	0,17500	9	0,27000
9	0,34375	10	0,10750
10	0,18125	11	0,27000
11	0,22500	12	0,05000
12	0,03125	13	0,05000
13	0,03125		
**D3S1358**		**vWA**	
15	0,09375	15	0,12750
16	0,33125	16	0,10750
17	0,35000	17	0,19500
18	0,20625	18	0,22250
19	0,01875	19	0,19500
		20	0,10250
		21	0,05000
**FGA**		**D13S317**	
19	0,03750	8	0,43750
20	0,08750	9	0,25625
21	0,35625	10	0,06250
22	0,22500	11	0,23750
23	0,05000	12	0,00625
24	0,24375		
**D5S818**		**D16S539**	
8	0,02500	8	0,13125
9	0,21250	9	0,16875
10	0,25625	10	0,29375
11	0,32500	11	0,28125
12	0,14375	12	0,08750
13	0,03750	13	0,03750
**CSF1PO**		**D18S51**	
8	0,23250	14	0,13750
9	0,35750	15	0,31875
10	0,20250	16	0,34375
11	0,15750	17	0,20000
12	0,05000		
**D8S1179**		**D21S11**	
9	0,02500	28	0,00625
10	0,08750	29	0,12500
11	0,23125	30	0,16875
12	0,50000	31	0,64375
13	0,15625	32	0,05625
**D7S820**			
8	0,25000		
9	0,38125		
10	0,05625		
11	0,3125		

## Results

The measurement of DNA contents and DNA purity examination of the extracted DNA samples results in the average value of DNA contents of **675 ± 5.35 ng/μl** with a purity range of **1.05-1.86**.

The PCR amplification was carried out through 13 STR CODIS loci. The visualization of PCR results was carried out through Polyacrylamide Agarose Gel Electrophorese (PAGE) with silver nitrate staining. The image of PCR results visualization is presented in Figure 1 below:

Table 1 above shows that 50 % of the genetic materials of an individual are inherited from each side of the parents. Since the nuclear DNA (nDNA) of an individual is inherited from both parents, it can be said that the inheritance of nuclear DNA follows the Mendelian way. The First Mendelian Law (Segregation of allelic genes) states the principles of allelic segregation during gamete formation. Gamete formation occurs through meiosis where the homologous pairs are decoupling and segregated. The alleles of a gene freely segregate from diploid to haploid (Elvita A et al., 2008: Mangoendidjojo W, 2014: Artadana IBM et al., 2018).

This finding indicates that the percentage of male-male siblings is 24%; female-female siblings is 36%, and male-female siblings is 40% (Figure 2 and Figure 3). Meanwhile the alleles of STR CODIS loci with the highest percentage are: TPOX allele 9 [34.375%], D3S1358 allele 17 [35%], FGA allele 21 [35.625%], D5S818 allele 11 [32.5%], CSF1PO allele 9 [35.75%], DS820 allele 9 [38.12%], D8S1179 allele 12 [50%], THOI allele 9 [27%], vWA allele 18 [22.25%], D13S317 allele 8 [43.75%], D16S539 allele 10 [29.375%], D18S51 allele 16 [34.375%], and D21S11 allele 31 [64.375%] (Table 2).

**Figure 6:**
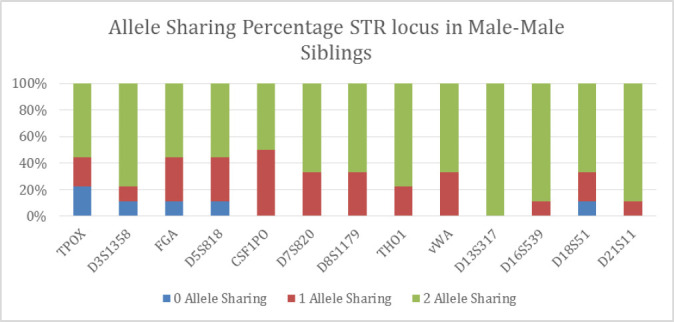
Percentage of STR loci allele sharing on male-male siblings

**Figure 7:**
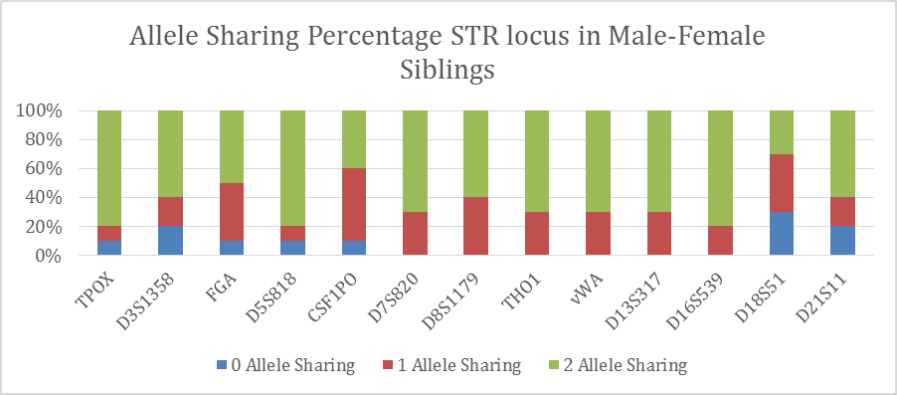
Percentage of STR loci allele sharing on male-female siblings

Figure 4 shows that D7S820, D8S1179, THO1, vWA, D13S317, and D16S539 with zero allele sharing are found in the samples while the highest percentage is found on two alleles sharing of TPOX and vWA loci (80%). In Figure 5 describing female-female siblings, the percentage of two alleles sharing at D21S11 locus is 85 %, and there is 0 (zero) allele sharing on all the examined loci. The male-male siblings’ group shows that D13S317 locus has two alleles sharing (100%) while CSF1PO, D7S820, D8S1179, THO1, vWA, D16S539, and D21S11 loci have 0 (zero) allele sharing (0%) (Figure 6). In male-female siblings, the loci D7S820, D8S1179, THO1, vWA, D13S317, and D16S539 with zero allele sharing is 0% (Figure 7).

The calculation of the Sibship Index (SI) of this study is presented in Table III below:

**Table 3:** Sibship Index Calculation

Sibship indices (SI) Probability Ratio	Strength	Percentage
< 1	Weak	10%
1 - 10	Moderate	10%
10 -100	Strong	25%
>100	Very Strong	55%

Table III displaying the result of sibling indices (SI) calculation shows that 55% of sibling pairs have SI higher than 100 (very strong) while 25% of sibling pairs have SI 10-100 (strong).

## Discussion

This study indicates the basic principles applied in assessing the probability of general alleles in the examined sample and inherited reference of Identical by Descent (IBD) alleles. There is a 25% probability that two siblings inherit two IBD alleles from their parents, a 50% probability that the siblings inherit one IBD allele and a 25% probability that the siblings do not inherit any IBD allele from the parents. There is no shared allele on the diploid loci. The probability that the two siblings do not share any alleles (25%) at one of the two loci might cause significant issues in identifying individuals who die in mass disasters if there is only one biological sibling alive. The probability that one sibling inherits zero IBD allele from their parents is constant at 25% for every locus. The probability that two biological siblings do not share alleles/partial loci depends on polymorphism information contents (PIC) and heterozygosity (Lee et al., 2012).

In genetics, alleles are the alternative forms of genes on specific loci that are related to the expression of certain characteristics (phenotype). Alleles are formed as the result of variations in nitrogen base sequences caused by mutation. In an individual, allele pairs determine the genotype of the individual. The term ‘allele’ exists as the result of the use of the term *allelomorph* in Mendel’s *Principles of Heredity* (Butler 2006: Elvita A et al., 2008: Butler et al., 2012).

Individuals with the same alleles at a locus are called to have a homozygote genotype while individuals with different alleles are heterozygote genotypes. Since genotypes are expressed into certain phenotypes, alleles may cause different appearances among individuals within a population (Butler 2006: Butler 2015).

In kinship examination using biological siblings, the sensitivity of variations depends on certainty thresholds. The higher the certainty threshold, the lower the sensitivity. High sensitivity is when the number of false-negative is low while high specificity is when the number of false-positive is low. Although biological siblings do not necessarily have the same alleles, allele sharing in biological siblings can be used to examine whether the siblings have kinship relationships as indicated in the study (Tzeng et al., 2000).

Small variations in allelic distribution between siblings are also affected by endogamous marriage where the authors recommend the use of 25 loci for the Lebanese population (Setyowati et al., 2020). In forensic cases such as the identification of fire disaster victims, siblings are compared with the victims by utilizing the Y-chromosome to identify whether the siblings and the victims come from the same father (Maeda et al., 2015). Many previous studies have been conducted to assess the relationship between biological siblings mainly based on heterozygosity and combined sibship indices (CSIs) (Reid et al., 2004). A study utilizing STR to analyze genetic diversity in Indonesia has been conducted to examine the extent of genetic mixing between Javanese and Arab ethnic groups (Sari, 2017).

A paternity test is a DNA test aimed to determine whether a man is the biological father of a child. Family dispute cases, in the form of doubting parents, are increasingly popular in Indonesian society (Syukriani Y, 2012).

Therefore, a paternity test uses parents as the comparison where the results are statistically close to 100% or about 99.99% (Untoro et al., 2009). The unavailability of information originating from a father, a mother, or a child that can be used as a comparison in forensic DNA examination becomes a problem in forensic DNA examination (Jacewicz et al, 2003). Unlike DNA testing with parental DNA as the comparison, the accuracy rate of personal identifications using siblings’ information is not close to 100% (O Connor, 2011).

The principle of identification examination through DNA is based on the process of allelic comparison between the victim or the perpetrator and the alleles from the family line, especially parents, following the Mendelian laws (Leclair B et al., 2004: Reid TM et al 2008: Hares DR, 2015). In case the parental or descendant line is unavailable, a comparison with a close family line is needed as a method taken in an identification examination through DNA, namely biological siblings.

The use of siblings as the comparison is one of the identification methods known as kinship analysis. Similar to a paternity test, the kinship analysis in an identification process also has a possibility of mismatched profiles in the examined DNA loci (O’Connor, 2011; Mangoendidjojo W, 2014; Maeda K et al., 2015; Marano LA et al., 2019; Sosiawan et al., 2019).

One of the aspects that need to be considered in assessing sibling relationships is minimizing the number of false-positive and increasing the number of the examined loci. The findings of this study show that male-male siblings of the Madurese population have a high percentage of allele sharing at D13S317, D16S539, and D21S11 loci while the female-female siblings at TPOX and D21S11 loci, and male-female siblings at TPOX, D5S818, vWA, D7S820, THO1, vWA, and D13S317 loci. These findings are beneficial in enriching the literature and the analysis of sibling relationships in Indonesian ethnic groups related to the paternity examination process or the identification of mass disaster victims.

## Conclusion

This study signifies that two alleles sharing is stronger compared to one-allele sharing and zero-allele sharing. The 50% of two alleles sharing indicates that the interpretation of full siblings is accepted so that the relationship claim is true. When assessing full sibling relationships, careful consideration should be given to STR loci with high frequencies of two alleles sharing. The findings of this study show that male-male siblings of the Madurese population have a high percentage of allele sharing at D13S317, D16S539, and D21S11 loci while the female-female siblings at TPOX and D21S11 loci, and male-female siblings at TPOX, D5S818, vWA, D7S820, THO1, vWA, and D13S317 loci. The main STR loci recommended for male-female siblings of the Madurese population are TPOX, D13S317, and D21S11.

### Conflict of Interest:

The authors declare that there is no conflict of interest in this study.

**List of Abbreviations:**
STR –: Short Tandem Repeat
DNA –: Deoxyribonucleic Acid
CODIS –: Combined DNA Index System
PCR –: Polymerase Chain Reaction
Amel–: Amelogenin
PAGEs -: polyacrylamide agarose gel electrophoreses
SI -: Sibship Indices
